# The Apollo Structured Vocabulary: an OWL2 ontology of phenomena in infectious disease epidemiology and population biology for use in epidemic simulation

**DOI:** 10.1186/s13326-016-0092-y

**Published:** 2016-08-18

**Authors:** William R. Hogan, Michael M. Wagner, Mathias Brochhausen, John Levander, Shawn T. Brown, Nicholas Millett, Jay DePasse, Josh Hanna

**Affiliations:** 1 University of Florida, P.O. Box 100219, 2004 Mowry Rd, Gainesville, FL 32610-0219 USA; 2 University of Pittsburgh, 5607 Baum Boulevard, Room 434, Pittsburgh, PA 15206 USA; 3 University of Arkansas for Medical Sciences, 4301 W. Markham St. Slot #782, Little Rock, AR 72205 USA; 4 University of Pittsburgh, 5607 Baum Boulevard, Room 434G, Pittsburgh, PA 15206 USA; 5 Pittsburgh Supercomputing Center, 300 S. Craig St., Pittsburgh, PA 15213 USA; 6 University of Pittsburgh, 5607 Baum Boulevard, Room 435 J, Pittsburgh, PA 15206 USA; 7 University of Florida, P.O. Box 100212, Gainesville, FL 32610-0212 USA

**Keywords:** Disease transmission model, Epidemic simulator, Epidemic simulation, Biomedical ontology, Infectious disease epidemiology, Population biology, Infection

## Abstract

**Background:**

We developed the Apollo Structured Vocabulary (Apollo-SV)—an OWL2 ontology of phenomena in infectious disease epidemiology and population biology—as part of a project whose goal is to increase the use of epidemic simulators in public health practice. Apollo-SV defines a terminology for use in simulator configuration. Apollo-SV is the product of an ontological analysis of the domain of infectious disease epidemiology, with particular attention to the inputs and outputs of nine simulators.

**Results:**

Apollo-SV contains 802 classes for representing the inputs and outputs of simulators, of which approximately half are new and half are imported from existing ontologies. The most important Apollo-SV class for users of simulators is *infectious disease scenario*, which is a representation of an ecosystem at simulator time zero that has at least one infection process (a class) affecting at least one population (also a class). Other important classes represent ecosystem elements (e.g., households), ecosystem processes (e.g., infection acquisition and infectious disease), censuses of ecosystem elements (e.g., censuses of populations), and infectious disease control measures.

In the larger project, which created an end-user application that can send the same infectious disease scenario to multiple simulators, Apollo-SV serves as the controlled terminology and strongly influences the design of the message syntax used to represent an infectious disease scenario. As we added simulators for different pathogens (e.g., malaria and dengue), the core classes of Apollo-SV have remained stable, suggesting that our conceptualization of the information required by simulators is sound.

Despite adhering to the OBO Foundry principle of orthogonality, we could not reuse Infectious Disease Ontology classes as the basis for infectious disease scenarios. We thus defined new classes in Apollo-SV for host, pathogen, infection, infectious disease, colonization, and infection acquisition. Unlike IDO, our ontological analysis extended to existing mathematical models of key biological phenomena studied by infectious disease epidemiology and population biology.

**Conclusion:**

Our ontological analysis as expressed in Apollo-SV was instrumental in developing a simulator-independent representation of infectious disease scenarios that can be run on multiple epidemic simulators. Our experience suggests the importance of extending ontological analysis of a domain to include existing mathematical models of the phenomena studied by the domain. Apollo-SV is freely available at: http://purl.obolibrary.org/obo/apollo_sv.owl.

## Background

The science and practice of infectious disease epidemiology, like climate science, is increasingly reliant on computational simulation [1], which is performed by software applications known as *epidemic simulators*. The simulators require information about pathogens, host populations, rates of infection transmission, interventions, and the disease outcomes of infections [2]. Using this configuration information—which we refer to as an *infectious disease scenario—*a simulator’s algorithm computes the progression of one or more infections in one or more populations over time, under zero or more interventions. The result of this computation—the *output* of the simulator—is information on which decision makers can base policy or decisions about disease control.

The goal of our research for the past 4 years has been to increase the accessibility and ease of use of simulators to promote progress in the field of infectious disease epidemiology [3]. A key focus has been reducing the time and effort required to locate a simulator, access it, understand its characteristics, create an infectious disease scenario to configure it, run it, and analyze its output. As an example of the effort required, Halloran et al. spent 6 months creating a comparative study of three simulators [4]. Most of the effort was expended on representing the same scenario in the different configuration representations and then converting results into a common representation for comparisons. As an example of the syntatic and semantic differences among simulator configurations, to configure the FRED simulator version 2.0.1 [5] to simulate the closing of schools^1^ 3 days after some event occurs (such as influenza incidence reaching a particular threshold) one would place “school_closure_delay = 3” in its configuration file, whereas for FluTE version 1.15 [6] one would place “responsedelay = 3” in its configuration file (unlike FRED, this setting would also affect other interventions such as vaccination).

To address this problem, we are developing a *common representation* for simulator configuration and output that is capable of representing the configurations and output of infectious disease simulators [3]. We use an XML Schema Document (XSD) as our primary representation because the XSD language enabled us to represent the probabilistic, mathematical, and other non-ontological knowledge required for and generated by simulation. We inform the design of the XSD representation by formal ontological analysis of the domain of infectious disease epidemiology, with particular attention to the inputs and outputs of nine simulators. Our goal was for the XSD to have the capability to represent the configuration and outputs of not only these nine simulators, but also other existing and future simulators. We represent the results of this analysis in an OWL ontology—called the *Apollo Structured Vocabulary* or *Apollo-SV*.

Apollo-SV and XSD together can be understood as a hybrid approach to knowledge representation and reasoning as defined by Davis et al. in their seminal paper on knowledge representation [7]. In particular, Apollo-SV (1) controls the terminology used in the XSD, (2) is a source of human-readable definitions of the terms for users of the XSD, and (3) serves as a record of the ontological commitments made by the developers of the XSD.

Our hypothesis was that it is feasible to develop a common representation for the configuration and output of simulators that are diverse both in their internal representations and in the pathogens, modes of transmission, geography, and interventions that they model.

We previously reported our initial versions of the XSD and Apollo-SV (versions 1.0), as well as our creation of a set of Web services to transmit a common configuration to two simulators [3]. We use configurations compliant with the XSD to invoke simulators as part of these Web Services, but generated the OWL2 representation—Apollo-SV—as our core ontology.

In this paper, we describe new results from our subsequent ontological analyses of additional simulators and our updated understanding of simulator configurations that we incorporated into Apollo-SV version 3.0.1.

## Methods

Our method for the development of the common representation was formal ontological analysis with rapid implementation of the representation to configure simulators and feedback from the results of implementation into further analysis.

The next sections discuss our style of ontology development, the application in which the ontology is used, and the procedures and principles we followed in constructing the OWL ontology, Apollo-SV.

### “Gene Ontology Style” of ontology development

We developed Apollo-SV using what we refer to as the *Gene Ontology style* of ontology development and testing—or *GO style* for short. GO style is a method for ontology development that emphasizes participation of subject matter experts and frequent and early feedback to ontology developers generated from using the ontology in software applications. We adopted GO style because it was successful for the Gene Ontology and because our community of developers and users was similar in many respects.

A key strength of GO style—which the Gene Ontology Consortium cites as a factor in its success—is that a community of scientists, ontologists, artificial intelligence experts, and software developers all contribute in an egalitarian fashion to the ontology and its applications [8]. The team developing Apollo-SV comprises experts in infectious disease epidemiology, simulator and other software development, disease surveillance, medicine, biomedical informatics, medical terminologies, ontological engineering, artificial intelligence, and formal logic (the last one in the list helps to ensure that OWL2 axioms that define classes are correct). All these individuals have been actively engaged in development and review of Apollo-SV, and their feedback guides design decisions.

A second strength of the GO style of ontology development is its emphasis on early use of the ontology in applications, which identifies issues and generates rapid feed back into ontology development [9]. We discuss the application of Apollo-SV in the next section. Additional elements of the style, that have subsequently been adopted by the Open Biological and Biomedical Ontologies (OBO) Foundry as principles of ontology development, include creating textual definitions for each class and making the ontology publicly and freely available for community use, review, and input [8–10]. We discuss how we implemented these additional elements of the style, as well as additional OBO Foundry principles, in the section following application.

### The application in which the ontology is used

As stated previously, Apollo-SV serves as the repository for definitions and standard terminology for the Apollo XSD. The Apollo XSD in turn is used in a set of Web services.

**Fig. 1:**
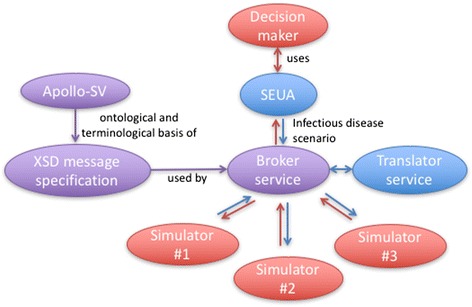
The relationships of Apollo components and epidemic simulators. Apollo-SV defines the terminology used in Apollo XSD, which specifies the message syntax for the Web services. The SEUA calls the Broker service to configure simulators (messages passed along *blue arrows*) and to access simulator output (messages passed along *red arrows*). The Translator service translates Apollo messages to/from native simulator input/output. *Purple* ovals represent Apollo standards; *blue* ovals represent Apollo-developed software that use the Apollo Web services; and *red* ovals represent entities interacting with Apollo

The Web services, called the Apollo Web Services, allow a publicly available, Web-based, end-user application to access multiple epidemic simulators through requests to a single Broker service (Fig. 1). In Fig. 1, the Simple End User Application (SEUA) [11] creates an infectious disease scenario for simulation, encoded in an XML document that conforms to the Apollo XSD syntax [12], which in turn uses terminology defined by Apollo-SV. The SEUA invokes the *runSimulation()* method of the Broker service with the XML-encoded infectious disease scenario. The Broker service subsequently invokes the Translator service, which translates the infectious disease scenario into the native terminology and syntax of the requested simulator(s). The SEUA polls the Broker service for the current status of the simulator until the status returned is “COMPLETED.” The SEUA then invokes various visualization services on the simulator output to display epidemic curves and maps in the interface.

By standardizing the terminology in the Web services, Apollo-SV helps to ensure that the SEUA end user and the simulators understand the XML-encoded infectious disease scenario to mean the same thing. Towards that end, the SEUA displays the textual definitions of classes in Apollo-SV to help the end user specify her infectious disease scenario accurately and precisely.

Beginning with the earliest development of Apollo-SV, exposing the terminology and definitions from Apollo-SV to subject matter experts, developers, and others in the SEUA was a significant source of critical feedback that led to additional ontological analysis as well as refinements of the terminology and definitions.

### Procedures and principles of Apollo-SV construction

We encode the results of our ontological analyses in OWL2. Our process proceeds concurrently with development of the Apollo XSD, and issues discovered in constructing either the OWL or the XSD are fed back into the analysis.

We conducted a formal ontological analysis of seven additional simulators—their configuration files, output files, documentation (including any user guides), and journal and conference papers that either described or used them. As part of this process, we reviewed terms that we extracted from these sources with the developers of the simulators to identify relevant but missing terms, to discover synonymy among terms, and to detect and resolve ambiguity. Of the seven additional simulators, four are presently connected to the Apollo Web Services.

We wrote a textual definition for every class that we create, in keeping with the GO style and OBO Foundry principles. We also created an elucidation annotation for classes in Apollo-SV because formal ontological textual definitions are sometimes not accessible to domain experts. The elucidation restates the definition in language more familiar to subject matter experts, while still referring to the same type of entities as the definition.

Also in accordance with the GO style of ontology development, we made Apollo-SV publicly available at [13], a permanent URL (PURL), to allow external scientific review, comments, and requests for additions as well as to encourage adoption of Apollo-SV. We ensured that Apollo-SV is easily accessible for browsing and download at the Web-based Ontobee portal [14], analogous to Gene Ontology browsers (the GO itself is viewable on Ontobee). The issue tracker is located at the Apollo GitHub site [15]. The PURL to the development version of Apollo-SV is at [16].

Because the Gene Ontology has “full membership” status in the OBO Foundry—a special status conferred on ontologies that conform to the OBO Foundry principles, we also followed the principles of the OBO Foundry in addition to openness and textual definitions [17, 18]. Per those principles, we release it in a common format, OWL2 [19].

We also adopted the Foundry principle of orthogonality, which stipulates that ontology developers reuse pre-existing ontological representations into Apollo-SV when and where appropriate.

We employed two methods for ontology reuse. The first method is the OWL2 ontology-import mechanism. This method inserts into the target ontology all classes and object properties of the imported ontology. However, bulk inclusion of large ontologies is often impractical and can degrade the usability of the target ontology. Therefore, the second method we used is the Minimum Information to Reference an External Ontology Term (MIREOT) methodology [20]. Using a MIREOT Protégé plugin that we developed [21], we import selected classes, individuals, and properties from certain ontologies into Apollo-SV.

We hypothesized that we would be able to reuse pre-existing ontologies or significant portions of them in developing Apollo-SV. In particular, we anticipated reusing substantial portions of the Infectious Disease Ontology (IDO) [22]. IDO is an OBO ontology (but not a “full member” of the Foundry) that represents infections, infectious diseases, pathogens, and hosts from the perspectives of infectious disease as a medical subspecialty and infectious disease research.

We adhered to OBO Foundry naming conventions [23]. We edited our terms to (1) avoid connectives (‘and’, ‘or’), (2) prefer singular nouns, (3) avoid the use of negations, and (4) avoid catch-all terms such as *Unknown x*.

To help link the OWL file to the XSD, we created a Unique Apollo Label (UAL) annotation for classes in Apollo-SV. The UAL is the exact XSD type or element name to which the class in Apollo-SV corresponds, for example, InfectiousDisease and BasicReproductionNumber.

Although not required by OBO Foundry principles, we imported Basic Formal Ontology (BFO) version 1.1 [24] into Apollo-SV as its upper ontology as do many other Foundry ontologies. The main reasons were (1) to maintain the semantics of BFO-based ontologies and their components that we reused and (2) to ensure that new classes and their associated axioms in Apollo-SV did not introduce inconsistencies to those semantics.

**Fig. 2:**
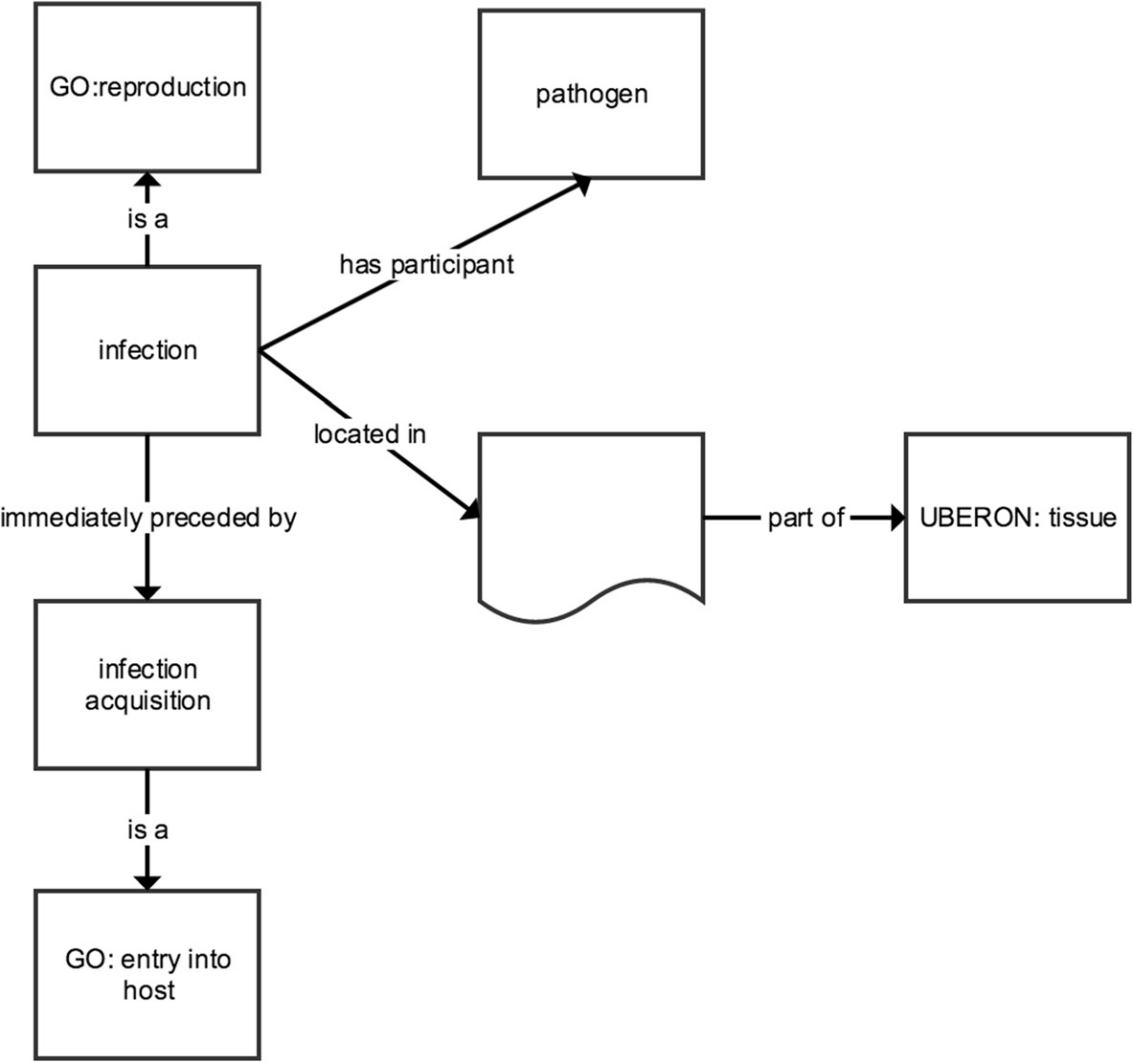
Representation of the equivalent class axiom for *infection* in Apollo-SV. *Boxes* represent named classes, *boxes* with *curved* bases represent anonymous classes, *arrows* represent object properties. In the *boxes* is the rdfs:label and the namespace of the source ontology, if different from Apollo-SV. Each *arrow* is labeled with the rdfs:label of the property it represents

**Fig. 3:**
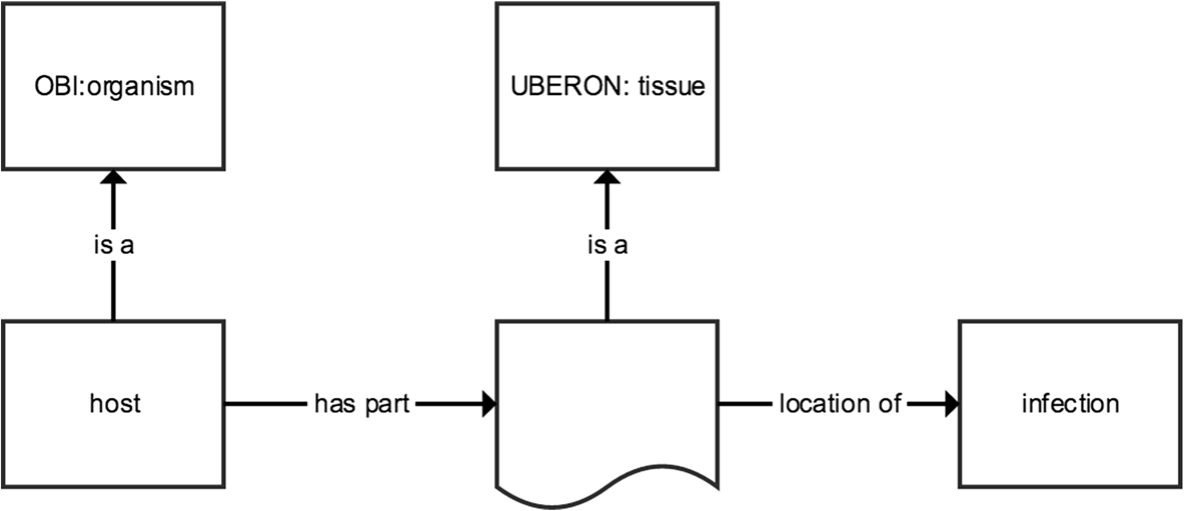
Representation of the equivalent class axiom for *host* in Apollo-SV. The graphical representation is analogous to Fig. 2

**Fig. 4:**
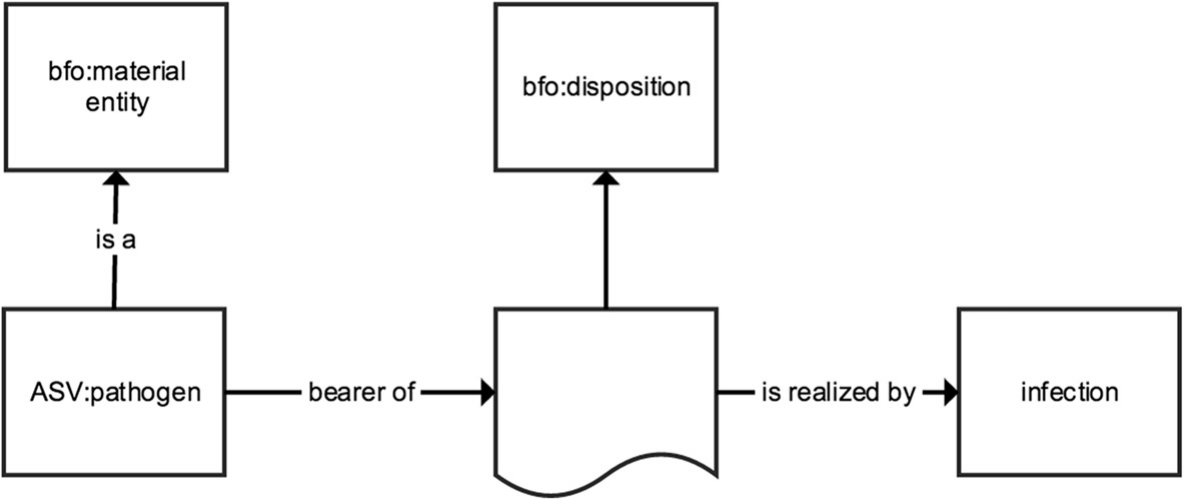
Representation of the equivalent class axiom for *pathogen* in Apollo-SV. The graphical representation is analogous to Fig. 2

**Fig. 5:**
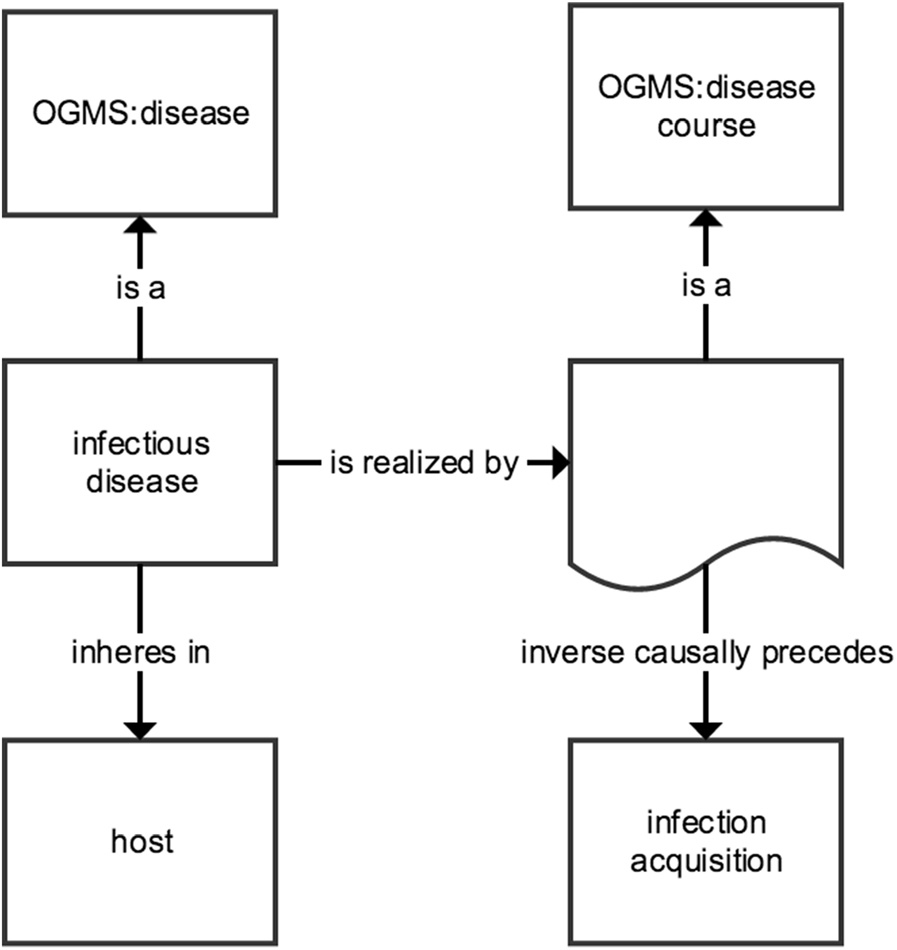
Representation of the equivalent class axiom for *infectious disease* in Apollo-SV. The graphical representation is analogous to Fig. 2

We created description logic axioms according to the syntax and semantics inherent in OWL2 for classes in Apollo-SV (e.g., Figs. 2,3, 4 and 5). When possible, these axioms provide both necessary and sufficient criteria for class membership. Many axioms, however, define only necessary criteria, most often because the description logic semantics of OWL2 were insufficiently expressive to encode both the necessary and sufficient criteria of the class.

## Results

Apollo-SV version 3.0.1 comprises 868 classes, of which 802 were required for describing simulator configuration and output. The remaining 66 classes are extraneous imported classes resulting from OWL2-based imports of ontologies in toto. Of the 802 classes, we created 397 (49.5 %) new classes, of which 117 classes have necessary and sufficient criteria. We imported 118 (14.7 %) classes via the methodology of Minimum Information to Reference and External Ontology Term or MIREOT (Table 1), and imported 287 (35.8 %) via OWL2-based import. The ontology comprises a total of 1180 logical axioms.

**Table 1:** Re-use of classes and object properties from pre-existing ontologies in Apollo-SV via MIREOT

Ontology	Classes	Object Properties	Total
Uberon	7	1	8
Ontology of Medically Related Social Entities	26	7	33
Gene Ontology	13	0	13
Ontology for General Medical Science	11	0	11
Ontology of Biomedical Investigations	21	6	27
Infectious Disease Ontology	3	7	10
The Drug Ontology	1	0	1
FlyBase Controlled Vocabulary	2	0	2
Vaccine Ontology	4	0	4
Drug-drug Interaction Evidence Ontology	1	0	1
Unit Ontology	5	0	5
Phenotypic Quality Ontology	3	0	3
Totals	97	21	118

### High level classes in Apollo-SV

The most important Apollo-SV class for users of simulators is *infectious disease scenario*, which represents an ecosystem at simulator time zero with at least one infection process (a class) affecting at least one population (also a class). The infectious disease scenario includes information about the infection process and its acquisition by a host organism (e.g., transmission probabilities and the durations of infectious and latent periods). It can also include information about planned or ongoing interventions to control infection (such as vaccination control measures). Representing ecosystems, populations, and censuses thus expanded the scope of Apollo-SV to population biology (Table 2). Including population biology subsequently influenced our definitions of key terms in infectious disease epidemiology.

**Table 2:** Classes in Apollo-SV by domain

Domain	Classes in Apollo-SV	
Infectious disease epidemiology	Infection	Infection acquisition
	Pathogen	Host
	Latent period	Infectious period
	Contaminated thing	Contamination acquisition
	Contamination	
	Infectious disease scenario	Basic reproduction number
	Transmission coefficient	Transmission probability
	Disease transmission model	Infectious disease control strategy
	Susceptible population	Exposed population
	Infectious population	Resistant population
Population biology	Ecosystem	Biotic ecosystem
	Abiotic ecosystem	Community
	Population	Population census
	Population infection and immunity census	Abiotic ecosystem census

Classes representing the infections, infection acquisitions, hosts, pathogens, and infectious diseases in an ecosystem are foundational in Apollo-SV. The reason is that the essential prediction of simulators is how many infections will occur given an infectious disease scenario. Nearly everything else that simulators predict are events that revolve around infection. They either (1) occur downstream of infection (such as disease outcomes including symptoms and death), (2) influence the probabilty of acquiring an infection (such as going to work or school or being vaccinated), or (3) occur as part of an infectious disease control strategy to prevent infection acquisition (such as school closure or quarantine). Also, because one simulator that we analyzed predicts colonization of hosts by pathogens and the processes by which hosts acquire colonizations, it was also important to represent colonization and how it differs from infection (see below).

### Foundational classes where reuse of IDO was not possible

We now describe a set of foundational classes we created in Apollo-SV after attempting unsuccessfully to reuse IDO classes and their definitions. We also discuss the reasons why these classes and definitions were unworkable.

#### Infection

Apollo-SV defines *infection* as: *A reproduction of a pathogen organism of a particular biological taxon in a tissue of a host organism from another taxon* (Fig. 2). From the perspective of population biology, an infection is merely a process by which one species reproduces, surviving from generation to generation, utilizing the resources of a host species. It is the normal biology of the pathogen species.

Infection is distinguished from other types of pathogen reproduction in a host—namely colonization (defined below)—by violation of the integrity of tissue in the host through tissue invasion. This tissue invasion may occur—and subsequently end—without causing any symptoms or permanent ill effects on the host. Thus, infection does not equate to disease, and we carefully distinguish between infection and infectious disease.

Epidemic simulators represent infection as a process because infectious disease epidemiologists define *infection* as a process. For example, [25, 26] define *infection* as the invasion of a host organism’s tissue by pathogens, the multiplication of those pathogens, and the reaction of the host’s tissue(s) to the pathogens and the toxins they produce. Further reinforcing the fact that infection is a process is the fact that simulators represent periods of (or ontologically speaking, *occurrent parts of*) the infection: the latent period and the infectious period.

Before we created a class for infection in Apollo-SV, we reviewed IDO for a class that represents the process of infection, whether labeled as *infection* or with some other term.

We found that IDO defines *infection* as a physical thing, or “material entity” in the terminology of Basic Formal Ontology (BFO). Specifically, it defines *infection* as: *A part of an extended organism that itself has as part a population of one or more infectious agents and that is (1) clinically abnormal in virtue of the presence of this infectious agent population, or (2) has a disposition to bring clinical abnormality to immunocompetent organisms of the same Species* [sic] *as the host (the organism corresponding to the extended organism) through transmission of a member or offspring of a member of the infectious agent population.*

Given that epidemic simulators and the relevant basic sciences on which they are founded recognize infection as a process, we needed to create a new class in Apollo-SV to represent it. The lack of a representation of the process of infection in IDO is surprising because IDO’s definitions of its classes *host role* and *infectious agent role* require a process to realize them. This process would presumably be infection.

#### Colonization

Apollo-SV defines colonization as: *A reproduction of a pathogen of a particular biological taxon inside or on the surface (*e.g.*, skin, mucosal membrane) of a host organism of another taxon, without invasion of any tissues of the host*. We required this class to represent the input of the Regional Healthcare Ecosystem Analyst [27] simulator, which models the spread of methicillin-resistant *Staphylococcus aureus* (MRSA). MRSA, as well as methicillin-sensitive varieties of *S. aureus*, typically colonize the nasal mucosa and skin of humans, living on these surfaces but not invading them. If a human host subsequently becomes immunocompromised or suffers a breach of the integrity of these surfaces, this colonization may extend to infection. Colonization is an important epidemiological process because an individual may acquire colonization from another MRSA colonized host.

IDO defines *colonization* as *An establishment of localization in host process in which an organism establishes itself in a host*. The latter part of the definition is more general than the former (assuming that there are other types of establishment besides localization) and thus does not differentiate this IDO class from its parent in IDO. We did not consider it further.

#### Host

Apollo-SV defines host as: *An organism of a particular biological taxon that is the site of reproduction of an organism of a different taxon* (Fig. 3). This definition accomodates the host undergoing infection and/or colonization. We note that our use of *site of* in this definition has a precise meaning as specified in the Relation Ontology, where *site of* is a synonym for the *contains process* relation, which relates an *…independent continuant and a process, in which the process takes place entirely within the independent continuant*.

We could not reuse IDO’s definition of *host*, which is: *An organism bearing a host role.* To understand this IDO definition, it is necessary to review two additional IDO definitions:

1. *Host role*: A *role borne by an organism in virtue of the fact that its extended organism contains a material entity other than the organism*.
2. *Extended organism*: *An object aggregate consisting of an organism and all material entities located within the organism, overlapping the organism, or occupying sites formed in part by the organism*.

Under these definitions, any organism that has an artificial joint, a penny in its gut, or an arrow through its chest is a host. Classifying a person with a prosthetic knee as a “host” is counterintuitive and not in keeping with how *host* is defined in population biology or infectious disease epidemiology (or in clinical medicine). Furthermore, the definition is based on IDO’s view of infection as a material entity and does not account for the process of infection.

#### Pathogen

Apollo-SV defines pathogen as: *An organism of a particular biological taxon that is the bearer of a disposition that is realized as its reproduction in the tissue of an organism of a different biological taxon* (Fig. 4). Thus Apollo-SV defines a pathogen as an organism that has the capability to reproduce inside the tissue of a host organism of another biological taxon. Note that this definition is inclusive of organisms like MRSA involved in colonization: the organism still has the *potential* to invade tissue and establish infection and thus meets the definition.

Once again, we had intended to reuse IDO. However, IDO defines *pathogen* as: *A material entity with a pathogenic disposition.* Again, this definition requires additional IDO definitions to clarify its meaning:

1. *Pathogenic disposition*: *A disposition to initiate processes that result in a disorder.*
2. *Disorder*: *A material entity which is clinically abnormal and part of an extended organism. Disorders are the physical basis of disease.*

Thus, per IDO any material that causes injury is a pathogen, including the endotoxin of *Clostridium difficile* or an overdose of acetaminophen. This definition is not how infectious disease epidemiology uses the term *pathogen*. IDO does have a class *infectious agent* as a subtype to *pathogen* that refers specifically to organisms that can enter into a host and cause disease. The IDO definition of infectious agent, however, relies on IDO’s definitions of *infection* and *infectious disorder* as material entities. To be consistent with infection as a process, we created the above definition of *pathogen* in Apollo-SV.

#### Infectious Disease

Apollo-SV defines *infectious disease* as: *A disease that inheres in a host and is realized as a disease course that is causally preceded by an infection* (Fig. 5). This means that the infection occurs first and creates abnormalities in the host that result in disease.

This definition is compatible with the OBO Foundry definition of *disease* in the Ontology of General Medical Science (OGMS) [28]. We thus were able to reuse the OGMS definition of *disease*, in keeping with the Foundry principle of orthogonality. Note that the disease inheres only in the host. From the pathogen’s perspective, there is no clinical abnormality (which is a necessary condition to meet the definition of disease in OGMS) as infection is normal biology of pathogens.

IDO’s definition of *infectious disease* is incompatible with our definition of *infection* as process.

#### Infection Acquisition

Apollo-SV defines *infection acquisition* as: *The biological process of a pathogen of a particular biological taxon entering (the tissues of the body of) a susceptible host organism of another taxon and reproducing using host resources*. A susceptible host can acquire an infection from one of at least three routes:

1. From another host organism (of the same or different species) that is infectious, which we represent in Apollo-SV as the class *Infection acquisition from infectious host*.
2. From some object or its surface that is contaminated with the pathogen, which we represent in Apollo-SV as the class *Infection acquisition from contaminated thing*.
3. From self colonization with the pathogen, which we represent in Apollo-SV as the class *Infection acquisition from self colonization*.

Note that we chose to define *infection acquistion* instead of *transmission* or *transmission process*. One reason was our insight that ontologically it is only the second, susceptible host that undergoes change during the process, and the term *infection acquisition* describes this change better than the term *transmission*. Another reason is that we needed to represent the acquisition of infections from contaminated things and from self-colonization with a pathogen. In both cases, transmission from host to host is indirect (mediated through contaminated surfaces and objects and through acquistion of colonization, respectively).

As with other key terms, IDO lacked an adequate class and definition for the process of infection acquisition. IDO imports *transmission process* and its two definitions from the Transmission Ontology:

1. *A process that is the means during which the pathogen is transmitted directly or indirectly from its natural reservoir, a susceptible host or source to a new host.*
2. *Suggested definition: A process by which a pathogen passes from one host organism to a second host organism of the same Species* [sic]*.*

Beginning with the second definition (which for some reason the Transmission Ontology labels as a “suggested definition”), it erroneously restricts transmission to occur only between two hosts of the same species. It is thus not usable in infectious disease epidemiology or any other science that studies cross-species transmission, which frequently occurs in zoonoses and diseases like foot and mouth disease.

The first definition has two major problems. The first problem is circularity, defining *transmission process* in terms of a pathogen being transmitted, with no definition of *transmitted*. The definition also excludes infection acquisitions from contaminated objects and self colonization and refers to the undefined terms *natural reservoir* and *source*.

The second problem is an ontological one. It attributes to one process the property of being the means by which something else happens. For example, assume droplet spread of infection from one host to another by a sneeze. This definition equates the sneeze with the transmission process. That is, it says that only the sneeze exists, but it also has the property of “having transmitted the pathogen”. However, equating the sneeze to the transmission process is nonsensical because for example, droplets can remain airborne and infectious for hours. Thus the pathogen may not reach (or be transmitted to) another host until long after the sneeze is over. The sneeze cannot therefore be the transmission process. In reality, there are two distinct processes: the sneeze and the subsequent acquisition of an infection by the second host.

### Testing Apollo-SV and its ontological commitments in software

We created a capability to configure six simulators: using the SEUA, an end user creates an infectious disease scenario that conforms to the XSD and then submits it to the simulators via Web services. The SEUA then retrieves the output of the simulators and displays it on maps and graphs. This capability was the end product of iterative, concurrent development of Apollo-SV and the XSD according to our analysis of the simulators, which included feedback from implementation in the Web services and SEUA. In addition, the SEUA displays textual definitions of Apollo-SV classes to the end user. Feedback on these definitions was fed back into ontology development which resulted in ontology changes including improved definitions. We are piloting a 7th simulator whose unique ontological commitments are reflected in Apollo-SV and the XSD, but are still undergoing refinement. The six configurable simulators are (1) a compartmental model developed by authors MMW, NEM, and JDL (disease agnostic); (2) the FRED model developed by the University of Pittsburgh Public Health Dynamics Laboratory in collaboration with the Pittsburgh Supercomputing Center Public Health Applications group and the School of Computer Science at Carnegie Mellon University (influenza A in humans); (3) the FluTE model developed by the University of Washington and Fred Hutchinson Cancer Research Center in Seattle (influenza A in humans), (4) a compartmental model of anthrax developed by authors MMW, NEM, and JDL, (5) the Computational Arthopod Agents (CLARA) dengue model developed by the Pittsburgh Supercomputing Center Public Health Applications group [29], and (6) an ebola model by Bellan et al. [30] These simulators are diverse in terms of underlying model (compartment vs. agent-based), disease (influenza, anthrax, ebola, and dengue), transmission (vector and person to person), and geography, both in terms of granularity (tract vs. county vs. entire nation) and scale (from a single state or nation to the entire globe).

## Discussion

We developed and implemented a common representation for simulator configuration and output and used it in an application that constructs and sends infectious disease scenarios to six different epidemic simulators. Our success in representing the inputs of a diverse sample of simulators lends support to our hypothesis that a common representation is feasible. Early usage of the ontology and exposure of its definitions to subject matter experts in software resulted in ontology improvements, most notably in the definitions of the core classes of Apollo-SV that we discussed here. This result is consistent with those of other ontology development efforts.

The ontological analysis we used to create the common representation identified abstractions that spanned simulators diverse in their core mathematical foundations (compartmental vs. agent based), pathogens, routes of transmission, geographical scope (single city or county vs. entire world), and interventions. The key abstractions were that the input of a simulator was an infectious disease scenario and that the scenario was properly understood as a representation of an ecosystem at a particular time, which corresponded to simulator time zero. We note that there is nothing specific to infectious disease in this conceptualization, which suggests that the ontology could be applied to simulation of other ecological phenomena.

A novel aspect of our method was its focus on the ontological analysis of epidemic simulators. This focus quickly brought into view the key biological phenomena being simulated and their fundamental nature. Additionally, simulators—being mathematical models—make explicit ontological commitments about the core entities involved in infections and their acquisition, which led us to confront the issues involved in representing them from the outset. It is worth noting that simulators used in epidemiology are often rigorously vetted through peer review of simulator-based research, as well as peer review of the simulators themselves. A final advantage of our focus on simulators is that they make a relatively small number of ontological commitments, which allowed us to devote sufficient time to them, while still being able to implement an application that continously tested whether the evolving representation could configure an expanding set of simulators. We expect that ontological analysis of any domain for which mathematical models exist would benefit from a focus on the models. For example, for human physiology there is an extensive library of mathematical models that are the focus of the Human Physiome project [31].

Prior work on the use of ontologies for modeling and simulation identified a distinction between so-called “referential” and “methodological” ontologies [32]. The former correspond with domain ontologies: a representation of the phenomena simulated. The latter correspond with application ontologies: a representation of simulators, how they work, and parameters that specify their operations. Apollo-SV is both a domain (a.k.a. referential) and an application (a.k.a. methodological) ontology in the field of infectious disease epidemiology.

We were surprised that we were unable to reuse classes from IDO for infection, pathogen, host, colonization, infectious disease, and transmission process. We conjecture that IDO’s ontological analysis may have begun with a disease focus and worked from there to the nature of infection, whereas we began with a biological science perspective. Our focus differed fundamentally from IDO’s concentration on how the terms are used in clinical medicine. In particular, our focus led us to a requirement to represent the process of infection, including key parts of this process such as the infectious period, as opposed to the steady-state, material-entity view of IDO.

We note however that our definitions of *infection*, *pathogen*, *host*, and *infectious disease* do not conflict with how these phenomena are understood by clinical medicine and thus could be reused without difficulty by ontologies that support clinical applications. In fact, in the case of zoonoses and infections that result from a prior process of colonization, our representations are a marked improvement because our definition of *infection acquisition* permits cross-species transmission and infections resulting from self colonization, whereas IDO’s definition of *transmission process* does not. Also, our definition of *host* and *pathogen* are more consistent with their usage by infectious disease specialists.

We also could not reuse other prior work on ontologies that have overlap with Apollo-SV. This work includes the Epidemiology Ontology (EO) [33] and the Ontology for Simulation Modeling of Population Health (SimPHO) [34]. EO—like Apollo-SV—strives to meet Foundry principles [33]. However it, like IDO, also defines *infection* as a material entity. It erroneously defines infection acquisition as occuring only in humans and does not axiomatize its classes. Okhmatovskaia et al. do not define for SimPHO [34] any of the terms in Table 1. Further comparison is not possible because SimPHO is not publicly available for review/reuse.^2^

Given that simulator configurations require representing several kinds of knowledge including probabilistic and mathematical knowledge, it was not possible to use an OWL2 representation in the Web services to configure simulators. At present the application that creates infectious disease scenarios does not invoke any description-logic reasoning supported by the axioms in Apollo-SV. Nevertheless, we found it advantageous to create the OWL2 representation and reuse it at the lower level of information representation of XSD. However, in other work, our OWL2 representation (i.e., Apollo-SV) supports reasoning in our ontology-based catalog of infectious disease epidemiology (OBC.ide), which is a catalog of datasets, publications, grey literature, and simulators [35]. The OBC.ide search interface makes use of multiple OWL2 reasoning capabilities including the “is a” hierarchy, transitive roles such as part of, and role chaining. Adaptation of Apollo-SV to this purpose required no re-axiomatization of the classes discussed here.

Our future plans include expanding Apollo-SV and the XSD to cover additional simulators and types of information used in infectious disease epidemiology.

## Conclusions

Apollo-SV captures the output of our ontological analysis of the entities in reality represented by epidemic simulator configuration and output. It also supplies the standardized terminology used in epidemic simulator configuration and output, which also includes an XSD-based syntax and database schema. We validated Apollo-SV through use in a simple end-user application that enables analysts to specify an infectious disease scenario and submit it to one or more of six simulators. Our analysis of biologically-grounded epidemic simulators and our process of testing the ontology in software led to scientifically accurate definitions that we have found to be reusable across diverse simulators to date. When available, mathematical models of natural phenomena like epidemics are potentially useful starting points for ontology development.

## Abbreviations

Apollo-SV: Apollo structured vocabulary
BFO: Basic formal ontology
DTM: Disease transmission model
EO: Epidemiology ontology
GO: Gene ontology
IDO: Infectious disease ontology
MIREOT: Minimum information to reference an external ontology term
OBO: Open biological and biomedical Ontologies
OGMS: Ontology for general medical science
OWL 2: Web ontology language version 2
OWL DL: OWL description logic
PURL: Permanent Uniform resource locator
SimPHO: Simulation modeling of population health ontology
UAL: Unique apollo label
XML: eXtensible markup language
XSD: XML schema document

## Footnotes

1 Closing schools is one infectious disease control strategy that simulators study for the control of influenza epidemics.

2 We are unable to find any remaining links to SimPHO, and past links while we were doing the work were broken at the time.
